# Comparison of pasting properties measured from the whole grain flour and extracted starch in barley (*Hordeum vulgare* L.)

**DOI:** 10.1371/journal.pone.0216978

**Published:** 2019-05-29

**Authors:** Xiangyun Fan, Juan Zhu, Wenbin Dong, Yuandong Sun, Chao Lv, Baojian Guo, Rugen Xu

**Affiliations:** 1 Jiangsu Key Laboratory of Crop Genetics and Physiology / Key Laboratory of Plant Functional Genomics of the Ministry of Education / Jiangsu Key Laboratory of Crop Genomics and Molecular Breeding, Agricultural College of Yangzhou University, Yangzhou, China; 2 Jiangsu Co-Innovation Center for Modern Production Technology of Grain Crops, Yangzhou University, Yangzhou, China; 3 Institute of Food Crops, Jiangsu Academy of Agricultural Sciences / Jiangsu Collaborative Innovation Center for Modern Crop Production, Nanjing, China; University of Tasmania, AUSTRALIA

## Abstract

Pasting properties of barley starch are important characteristics from a processing standpoint. The isolation of starch form barley grains is time consuming thus the whole grain flour is always used. To compare pasting properties of starch with those of the whole grain flour, we used a Rapid Visco Analyser (RVA) to measure pasting properties of three types of samples: grain flour and starches isolated using two different extraction methods. We also investigated compositional, morphological and structural properties of the two starch samples. Significant differences in pasting properties were found among the three sample types, but most of the parameters of pasting properties displayed significant correlations between flour and starch. No significant differences were found in amylose/amylopectin ratio, granule morphology, granule size distribution and crystal structure between starches extracted using two different methods. However, the starch isolated from water homogenization had a higher protein content and lower total starch, amylose and amylopectin contents than the starch extracted with homogenized extraction under alkaline conditions. We concluded that the whole grain flour can be used to predict the pasting properties in breeding programs.

## Introduction

The functionality and quality of starchy cereal-based products mainly depend on starch properties and characteristics [1]. The pasting property is one of the important starch physiochemical properties and is affected by multiple factors [2–5]. Starch pasting properties are highly influenced by the composition proportion and structure of starch (e.g. total starch as well as amylose and amylopectin content, the ratio of amylose and amylopectin, the proportion of starch granules with distinct size, distribution of chain length) [5–6]. In addition, other components in the test sample such as protein, lipids, sugars, emulsifiers, salts, fatty acids and grain husk can also influence properties to varying degrees [2, 7–10].

Several different methods have been used for starch pasting analysis. These include Rapid Visco Analyser (RVA), rheometer and amylograph. Of these, RVA is the most commonly used method in the viscosity measurement, primarily because of its advantages in fast determination and the smaller sample sizes required [11]. In the malting and brewing process, starch is degraded by enzymes to provide substrates for the fermentative phase and gelatinization is essential for starch enzymatic hydrolysis during mashing [12]. Given that barley is the primary source for malting and brewing industries with starch being the major constituent in barley grain, changes in starch pasting characteristics have direct effects on grain malting properties [12] and RVA has become the instrument of choice for determining and evaluating grain and malt quality in barley [11, 13–16]. It has been reported that pasting properties have a close relationship with malting quality traits especially fine extract in barley, and a shorter time to peak viscosity and lower peak time are considered to be suitable for high malting and food processing [15–18]. However, unlike some other cereals such as rice or wheat, the hull of barley grain (about 13% by weight on average) is difficult to remove. Even after passing through the sieve after milling, a proportion of grain bran remains in barley flour. According to the report in rice, the bran which constitutes approximately 8–10% of the total grain and is rich in protein, lipid and fiber can have significant influence on flour pasting properties [9]. To accurately measure starch properties, starch should be isolated from the flour. Apart from enzyme-assisted extraction, two common methods are used for native starch extraction, water-homogenized isolation and homogenized extraction under alkaline conditions [19]. However, some studies have reported the influence of alkali on starch physicochemical and structural properties in mungbean and cassava, sago and native cereal starches [20–22].

Given that starch isolation is expensive and time consuming, can whole grain flour be used to predict starch pasting properties? The purpose of this study is to compare pasting properties of barley grain flour and extracted starches using two different methods. The results will provide a useful reference to researchers in selecting quality traits.

## Materials and methods

### Plant materials

Four Chinese barley (*Hordeum vulgare* L.) varieties were used in this study. Of them, Yangnongpi7 (YNP7) and Yangnongpi11 (YNP11) were hulled barley, while Emai507 (EM507) and Huangchangmang (HCM) were hull-less barley. In addition, the pasting properties of other thirty hulled barley varieties were also measured using RVA (S1 Table). All materials were planted at Yangzhou (32°24'N, 119°26'E) in 2017–2018 growing seasons with routine agronomic managements.

### Sample preparation

After harvesting, the mature grains were air-dried and milled into flour. Thereafter, the flours were passed through a 100-mesh sieve, dried at 45°C for 36 h and stored at 4°C in sealed plastic bags. Two extraction methods were used in the isolation of native starches. One (Method 1) was adopted as described by Li et al. [23] with slight modifications. Briefly, grains were sliced in four pieces by a blade and the grain pieces were further steeped in double-distilled water at 4°C for 48 h. The ratio of grain pieces to soaking solution was 1:5. Then, the softened grain pieces were homogenized with ice-cold water with a blender (IKA-T RCT-Basic, Germany). The slurry was filtered with 100-, 200- and 300-mesh nylon sieves and centrifuged at 4000 rpm for 20 min. The supernatant was discarded and the yellow gel-like layer on top of the packed white starch granule pellet was carefully scraped off and removed. The process of centrifugation separation was repeated three times. The precipitated starch was further repeatedly steeped and washed with anhydrous ethanol three times, dried at 45°C for 36 h, passed through the 100-mesh nylon sieve and stored at 4°C in sealed plastic bags. Another method (Method 2) was following the method described by Claver et al. [24] with slight modifications. Briefly, the grain flour was immersed in a 0.2% aqueous NaOH solution and kept at 4°C for 24 h. Before stepwise filtration through 100-, 200- and 300-mesh nylon sieves, the slurry was blended for 3 min. The filtrate was then centrifuged using freezing centrifugation at 4000 rpm for 20 min. The supernatant was discarded and the top yellow layer was carefully scraped off and removed. The process was repeated three times. The lower starch layer was repeatedly steeped and washed with anhydrous ethanol three times, dried at 45°C for 36 h, passed through the 100-mesh nylon sieve and stored at 4°C in sealed plastic bags.

### RVA analysis

RVA (RVA-3D, Newport Scientific, Narrabeen, Australia) was used in this study. 4.0 g flour or 3.0 g starch was directly weighed into an RVA canister, followed by the addition of 25 g distilled water. The starch slurry was heated from 50°C to 95°C at the rate of 12°C/min, maintained at 95°C for 2.5 min, and then cooled to 50°C at the same rate. Paddle speed was set at 160 rpm [23]. Parameters recorded were peak viscosity (PV), trough viscosity (TV), breakdown (BD), final viscosity (FV), setback (SB), peak time (PT) and gelatinization temperature (GT) [23]. Each analysis was repeated at least twice.

### Composition measurement

The total starch content, amylose content, amylopectin content and the ratio of amylose/amylopectin were determined by using Total Starch Assay Kit (K-TSTA, Megazyme International Ireland Ltd.) together with AM/AP assay Kit (K-AMYL, Megazyme International Ireland Ltd.). The protein content was measured by using FOSS Kjeltec ^™^ 2300 (Foss Analytical AB, Sweden) according to the Kjeldahl method [25] and the experiments were performed in triplicate.

### Morphology observation of starch granules

The starch was pretreated and then viewed with an environmental scanning electron microscope (SEM) (Philips XL-30) according to the method of Fan et al. [26].

### Starch granule size distribution

The particle size of the starch was analysed by a laser diffraction particle size analyser (Mastersizer 2000, Malvern, UK) according to the method of Fan et al. [26]. The starch samples were passed through the 300-mesh nylon sieve before measurement.

### ATR-FTIR analysis of starch samples

Ordered structure of starch external region was analysed on a Varian 7000 Fourier transform infrared (FTIR) spectrometer with a deuterated triglycine sulphate detector equipped with an attenuated total reflectance (ATR) single reflectance cell containing a germanium crystal (45° incidence angle) (PIKE Technologies, USA) as previously described by Cai et al. [27].

### Crystal structure analysis of starch samples

Crystal structure of starch was analyzed by X-ray diffraction (XRD) (D8, Bruker, Germany). The pretreatment of starch samples, XRD analysis and relative crystalline degree (%) analysis were performed by the method described by Fan et al. [26]. The relative crystallinity was quantitatively determined three times.

### Statistical analysis

Analysis of variance (ANOVA), multiple comparison, t-test and correlation analysis as well as descriptive statistical analysis were all conducted with SPSS statistical software Version 16.0.

## Results

### Comparison of pasting properties measured from flour and starch samples

**Fig 1:**
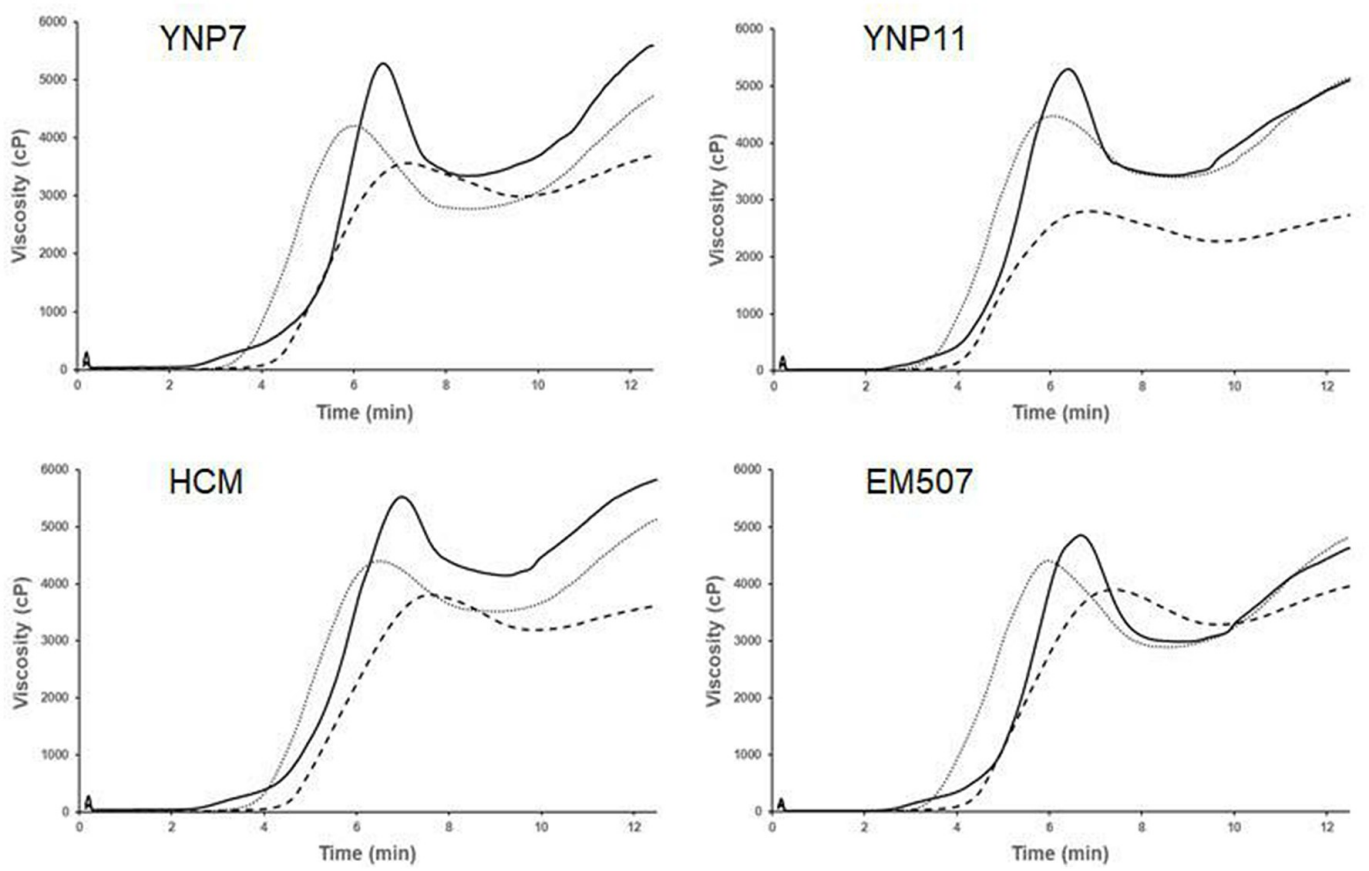
RVA profiles of different samples from four barley cultivars. —, ------, ∙∙∙∙∙∙∙ respectively represent the sample of grain flour, the sample of starch isolated from method 1, the sample of starch isolated from method 2.

The pasting properties of the three different samples from four barley varieties as analyzed by RVA are shown in Table 1, and the RVA profile is given in Fig 1. Significant differences in all seven RVA parameters existed among varieties and samples (Tables 1 and 2). The flour sample displayed the highest PV, TV, BD, FV and SB but the lowest GT. Starch extracted using method 2 had the shortest PT. Starch samples isolated from method 1 had the lowest PV, BD, FV and SB but the highest PT and GT (Table 1). Moreover, RVA profile parameters of 30 barley varieties were measured using grain flour and starch isolated from method 2 (S1 Table), and the descriptive statistics, correlation and difference analysis between the two samples are presented in Table 3. Again, grain flour showed significantly higher PV, BD, SB, GT and longer PT than starch samples. Significant differences were observed in PV, TV, BD, SB, PT and GT existed among varieties and samples (Tables 3 and 4). As shown in Table 3, PV, TV, BD, PT and GT of flour samples showed significant positive correlations with those of starch samples. Surprisingly, SB of flour samples showed a significant negative correlation with that of starch samples (Table 3).

**Table 1:** RVA profile parameters between three diverse samples of four barley varieties.

Varieties	Samples	PV (cP)	TV (cP)	BD (cP)	FV (cP)	SB (cP)	PT (min)	GT (°C)
YNP7	I	5280±38c	3342±27c	1938±65c	5591±27c	2249±54c	6.6±0.05b	71.2±1.09a
	II	3567±11a	2991±14b	576±25a	3693±16a	702±30a	7.2±0.05c	87.3±0.49c
	III	4205±17b	2775±28a	1430±11b	4710±10b	1935±18b	5.9±0.05a	77.3±1.09b
YNP11	I	5315±56c	3425±89c	1890±33b	5096±33c	1543±56b	6.3±0.09b	73.4±0.35a
	II	2801±13a	2274±31a	527±18a	2735±24a	461±55a	6.8±0.00c	84.9±0.53c
	III	4478±25b	3401±21b	1077±46b	5140±21b	1739±42c	6.1±0.05a	75.3±0.60b
HCM	I	5545±35c	4139±95c	1406±130c	5820±24c	1681±119b	7.0±0.09b	72.4±0.04a
	II	3804±15a	3186±13a	619±2a	3610±2a	424±11a	7.6±0.00c	89.8±0.67c
	III	4396±42b	3467±27b	929±15b	5119±18b	1652±45b	6.5±0.00a	81.7±0.60b
EM507	I	4858±23c	2990±26a	1869±49c	4629±4b	1640±22b	6.7±0.05b	71.6±1.09a
	II	3896±21a	3281±35b	615±14a	3949±28a	668±7a	7.4±0.05c	86.5±0.67c
	III	4399±14b	2887±34a	1512±48b	4810±43c	1923±9c	5.9±0.05a	75.7±0.04b

I, the sample of grain flour; II, the sample of starch isolated from method 1; III, the sample of starch isolated from method 2. Different letters are significantly different (P<0.05).

**Table 2:** ANOVA of RVA profile parameters of three diverse samples in four barley varieties.

	*df*	PV		TV		BD		FV		SB		PT		GT	
		*MS*	*F*	*MS*	*F*	*MS*	*F*	*MS*	*F*	*MS*	*F*	*MS*	*F*	*MS*	*F*
Variety(V)	3	88843.78	115.13**	650351.17	310.64**	260795.17	102.11**	417698.94	742.91**	155891.11	67.82**	0.58	210.02**	20.23	43.03**
Sample(S)	2	6118673.17	7929.00**	733085.04	350.16**	2810701.29	1100.00**	7233933.88	12870.00**	4080497.54	1775.00**	2.35	845.50**	389.95	829.31**
V×S	6	387420.94	502.06**	308850.21	147.52**	83901.46	32.85**	422381.82	751.24**	48637.65	21.16**	0.07	26.57**	11.73	24.94**
Error	12	771.67		2093.58		2554.08		562.25		2298.58		0.003		0.47	

**Indicate significant at 1% level (P < 0.01).

**Table 3:** Descriptive statistics, correlation and difference analysis of RVA parameters from thirty barley varieties between two samples.

	PV (cP)		TV (cP)		BD (cP)		FV (cP)		SB (cP)		PT (min)		GT (°C)	
	I	II	I	II	I	II	I	II	I	II	I	II	I	II
Mean	4552	4151	2857	2786	1690	1364	4431	4534	1573	1748	6.49	5.84	81.4	75.3
Minimum	3282	3225	2113	2313	1134	537	3299	3618	880	1089	6.00	5.20	69.15	71.65
Maximum	5568	5048	3669	3276	2312	2103	5305	5617	2240	2502	6.80	6.40	87.80	81.25
SD	641	407	416	258	341	345	580	445	332	295	0.21	0.27	5.86	2.56
Variance	411500	166400	172800	66530	116500	118900	337000	198500	110000	87280	0.044	0.075	34.33	6.528
*t*	3.842**		1.025		6.084**		-0.759		-2.075*		13.389**		6.336**	
*r*	0.480**		0.444*		0.633**		-0.380		-0.730		0.419*		0.424*	

I: the sample of grain flour; II: the sample of starch isolated from method 2; r: correlation between flour samples and starch samples.

*Indicates significant at 5% level (P < 0.05);

**Indicates significant at 1% level (P < 0.01).

**Table 4:** ANOVA of RVA profile parameters measured of flour and starch in thirty barley varieties.

	*df*	PV		TV		BD		FV		SB		PT		GT	
		*MS*	*F*	*MS*	*F*	*MS*	*F*	*MS*	*F*	*MS*	*F*	*MS*	*F*	*MS*	*F*
Variety	29	414505.18	2.54**	167276.85	2.32	192210.01	4.45**	257797.13	0.93	91478.68	0.86	0.08	2.36*	26.78	1.9*
Sample	1	2410812.15	14.76**	75686.02	1.05	1599360.27	37.01**	159753.6	0.58	455358.82	4.30*	6.36	179.28**	565.34	40.14**
Error	29	163311.81		72099.93		43214.92		277659.09		105798.51		0.04		14.08	

*Indicate significant at 5% level (P < 0.05);

**Indicate significant at 1% level (P < 0.01).

### Proximate chemical composition analysis

The data on composition properties of grain flour and the two starch samples from four barley varieties is presented in Table 5. In grain flour, protein contents were in the range of 11~14% and total starch contents as well as amylose and amylopectin contents were much lower than starch samples. No significant difference in the amylose/amylopectin ratio was found among grain flour and two starch samples (Table 5). The protein content in starch isolated from water-homogenized method (method 1) was slightly but significantly higher than that of starch isolated with sodium hydroxide (method 2) and total starch, amylose and amylopectin contents were lower in starch samples extracted using method 1 (Table 5).

**Table 5:** The composition characteristics of flour and two starches samples from four barley varieties.

Varieties	Samples	Starch (%)	Protein (%)	Amylose (%)	Amylopectin (%)	A/P
YNP7	I	58.81±0.83a	11.98±0.28c	15.27±0.44a	43.54±0.44a	0.35±0.01
	II	91.07±0.91b	0.60±0.04b	23.97±0.16b	67.09±0.16	0.35±0.00
	III	97.25±1.03c	0.30±0.05a	25.41±0.47b	71.85±0.47c	0.35±0.01
YNP11	I	62.61±0.61a	11.50±0.11c	16.11±0.56a	46.50±0.56a	0.35±0.00
	II	86.67±0.90b	0.73±0.04b	21.80±0.47b	64.86±0.47b	0.34±0.01
	III	98.79±0.52c	0.21±0.04a	24.91±0.65c	73.88±0.65c	0.34±0.01
HCM	I	65.64±1.23a	14.08±0.10c	17.63±0.63a	48.01±0.63a	0.37±0.01
	II	90.89±0.85b	0.57±0.04b	25.09±0.53b	65.81±0.53b	0.38±0.01
	III	96.00±0.82c	0.28±0.03a	26.57±0.63b	69.42±0.63c	0.38±0.01
EM507	I	57.86±1.08a	13.70±0.09c	14.88±0.76a	42.98±0.76a	0.35±0.01
	II	93.63±0.83b	0.53±0.05b	23.79±0.49b	69.84±0.49b	0.34±0.01
	I	98.76±0.74c	0.26±0.04a	25.11±0.38b	73.65±0.38c	0.34±0.01

I, the sample of grain flour; II, the sample of starch isolated from method 1; III, the sample of starch isolated from method 2. Different letters are significantly different (P<0.05).

### Morphology and size distribution of starch granules

**Fig 2:**
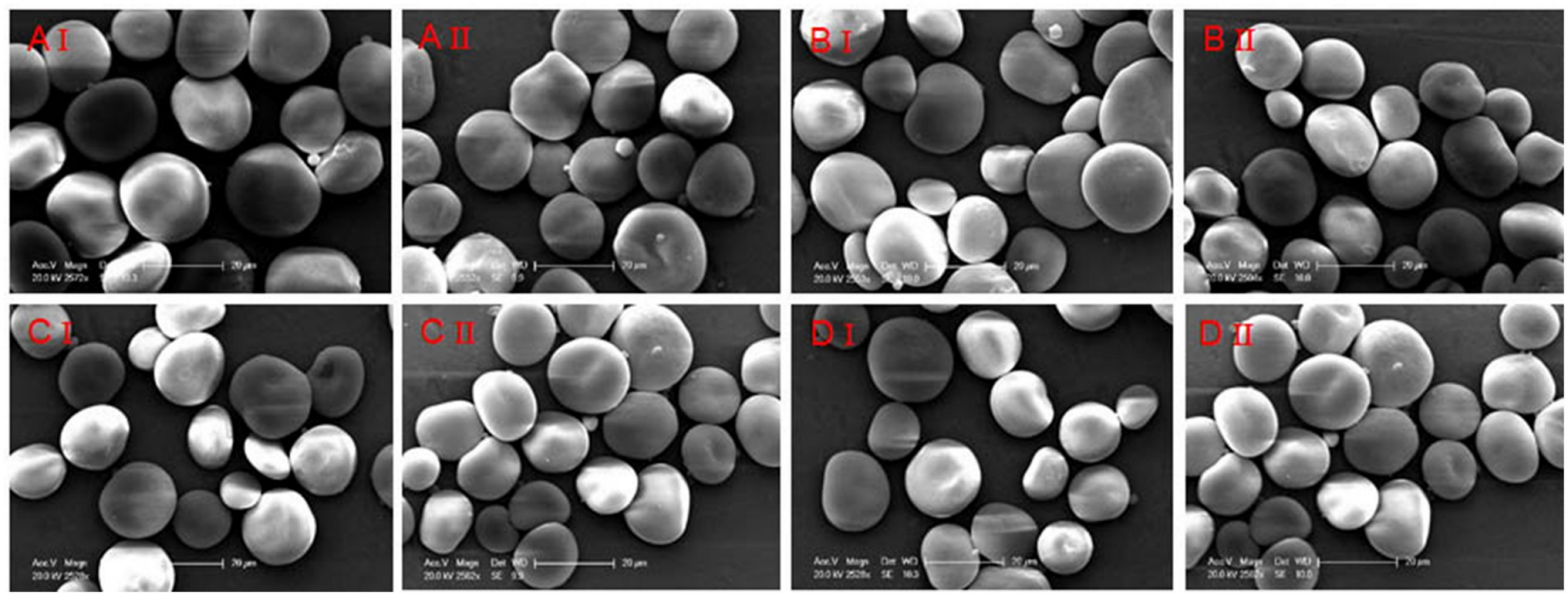
Scanning electron micrographs of two starch samples from four barley cultivars. A: YNP7; B: YNP11; C: HCM; D: EM507. I, the sample of starch isolated from method 1; II, the sample of starch isolated from method 2.

Scanning electron microscopy (SEM) images of starch granules isolated by two methods from four barley varieties are shown in Fig 2. Morphologically, all starch granules were similar in shape, size and surface. No obvious differences in the distribution of starch granule size and the morphological property were found between the two starch samples (Table 6; S1 Fig).

**Table 6:** The fine structure characteristics of starches isolated by two methods from four barley varieties.

Varieties	Samples	Average granule size (μm)	IR ratio		X-ray diffraction pattern	Relative crystallinity (%)
			(1045/1022) cm^-1^	(1022/995) cm^-1^		
YNP7	I	17.83±0.14	0.554±0.006	1.023±0.001	A	23.73±0.44
	II	17.55±0.16	0.561±0.008	1.024±0.006	A	24.23±0.30
YNP11	I	18.06±0.11	0.523±0.002	1.022±0.008	A	22.30±0.19
	II	18.00±0.07	0.519±0.007	1.030±0.005	A	22.91±0.78
HCM	I	17.18±0.09	0.570±0.009	1.046±0.011	A	24.51±0.49
	II	17.26±0.06	0.555±0.006	1.038±0.004	A	24.85±0.56
EM507	I	18.56±0.07	0.538±0.001	1.038±0.001	A	23.24±0.51
	II	18.42±0.14	0.545±0.009	1.042±0.006	A	23.59±0.61

I, the sample of starch isolated from method 1; II, the sample of starch isolated from method 2. Different letters are significantly different (P<0.05).

### XRD and ATR-FTIR analysis

The ratios of 1045/1022 and 1022/995 cm^−1^ of starch are summarized in Table 6, which serves as a convenient index of FTIR data in comparison with other measures of starch conformation. In the present work, based on both the spectra and calculated data, starches isolated from two methods showed no significant difference in the structure of starch external region (Table 6; S2 Fig). The X-ray diffraction (XRD) spectra of two starch samples from four barley varieties is presented in S3 Fig, and the relative crystallinity data are listed in Table 6. All of the starch samples exhibited typical A-type crystalline packing arrangements with strong reflections at 2θ of about 15°, 17°, 18°, 20° and 23° (Table 6; S3 Fig). The relative crystallinity of the starch sample isolated from water-homogenized method was similar compared with that of the starch sample isolated with sodium hydroxide (Table 6).

## Discussion

RVA has been widely used to determine starch pasting properties [11]. In barley, grain flour was usually used as the sample in RVA measurement to evaluate or predict grain and malting quality [11, 13–18] as the grain flour can be obtained easily and efficiently compared with extracted starch and the whole grain is the main and integral part in utilization and production [11]. However, there is no report on the correlation between RVA profile characteristics measured by grain flour and starch in barley. In starch extraction, wet-milling is the main process used to produce pure starch with high quality and yield [28]. The starch extraction method of water homogenization is widely used in cereals especially in rice. However, barley grain is rich in protein and soluble sugar, which can produce high viscosity in aqueous solutions and strong binding with starch [29]. Wu et al. [30] isolated starch with sodium hydroxide to eliminate the influence of β-glucan. As the alkali with particular concentration has been reported to affect starch pasting and some other properties in some native cereal starches [20–22], two methods, water-homogenized isolation and homogenized extraction under alkaline conditions, were used for starch extraction in this study. In the extraction process of water-homogenized isolation (method 1), the yellow gel-like layer is very hard to scrape off and remove completely because of the impurity layer is mixed with starch which is associated with the existence of β-glucan and protein, leading to relatively higher starch concentration than the samples extracted with NaOH (method 2). The fine structure measured by scanning electron microscope (SEM), laser diffraction particle size, attenuated total reflectance-fourier transform infrared (ATR-FTIR) and X-ray diffraction (XRD) analysis revealed no significant differences in these starch properties between two starch samples, suggesting that the fine structure of barley grain starch was not changed by 0.2% NaOH.

Other components in starch can influence the RVA analysis [2, 7–10], hence, different barley varieties with a different proportion of other components apart from starch in grain flour resulted in diverse changes in starch pasting properties measured by RVA. In our study, significant differences in RVA profile characteristics existed among varieties and samples. Even though viscosities of flour samples are significantly lower than those of starch samples due to lower starch contents in the flour samples, significant correlations were found for most RVA parameters (PV, TV, BD, PT and GT) between flour and starch, indicating that the whole grain flour can be used to predict the pasting properties of starch.

It is well known that lipid and soluble sugar can be removed by steeping and washing with anhydrous ethanol [23]. Significant differences in RVA measurements were also found between the two starch samples isolated from two different methods, with the water-homogenized isolated starch showing lower viscosity parameters but higher GT (Table 1). This result is consistent with the findings of Lai et al. [22] and Kaur et al. [31] and is mainly due to the higher concentration of starch in samples extracted with Method 2.

In conclusion, significant correlation was found between RVA measurements of grain flour and starch. Considering that grain flour is easier to be obtained, when a large number of samples need to be tested in breeding programs, the whole grain flour can be used. Barley grain starch isolated with 0.2% NaOH showed no changes in structural properties and is suggested to be used in studying starch properties.

## Supporting information

S1 Fig Starch granule distribution of two starch samples from four barley varieties. I, the sample of starch isolated from method 1; II, the sample of starch isolated from method 2.
(TIF)

S2 Fig ATR-FTIR spectra of two starch samples from four barley varieties. A: YNP7; B: YNP11; C: HCM; D: EM507. I, the sample of starch isolated from method 1; II, the sample of starch isolated from method 2.
(JPG)

S3 Fig X-ray diffraction spectra of two starch samples from four barley varieties. A: YNP7; B: YNP11; C: HCM; D: EM507. I, the sample of starch isolated from method 1; II, the sample of starch isolated from method 2.
(JPG)

S1 Table RVA profile parameters measured of grain flour and starch from thirty barley varieties. (DOCX)

## Abbreviations

ATR-FTIR: attenuated total reflectance-Fourier transform infrared
BD: breakdown
FV: final viscosity
GT: gelatinization temperature
PT: peak time
PV: peak viscosity
RVA: rapid visco analyser
SB: setback
SEM: scanning electron microscope
TV: trough viscosity
XRD: X-ray diffraction
