# Supplementary figures and images for: Comparison of pasting properties measured from the whole grain flour and extracted starch in barley (Hordeum vulgare L.)
Source: PLoS One. 2019 May 29;14(5):e0216978. doi: 10.1371/journal.pone.0216978 (PMC6541268; doi:10.1371/journal.pone.0216978)

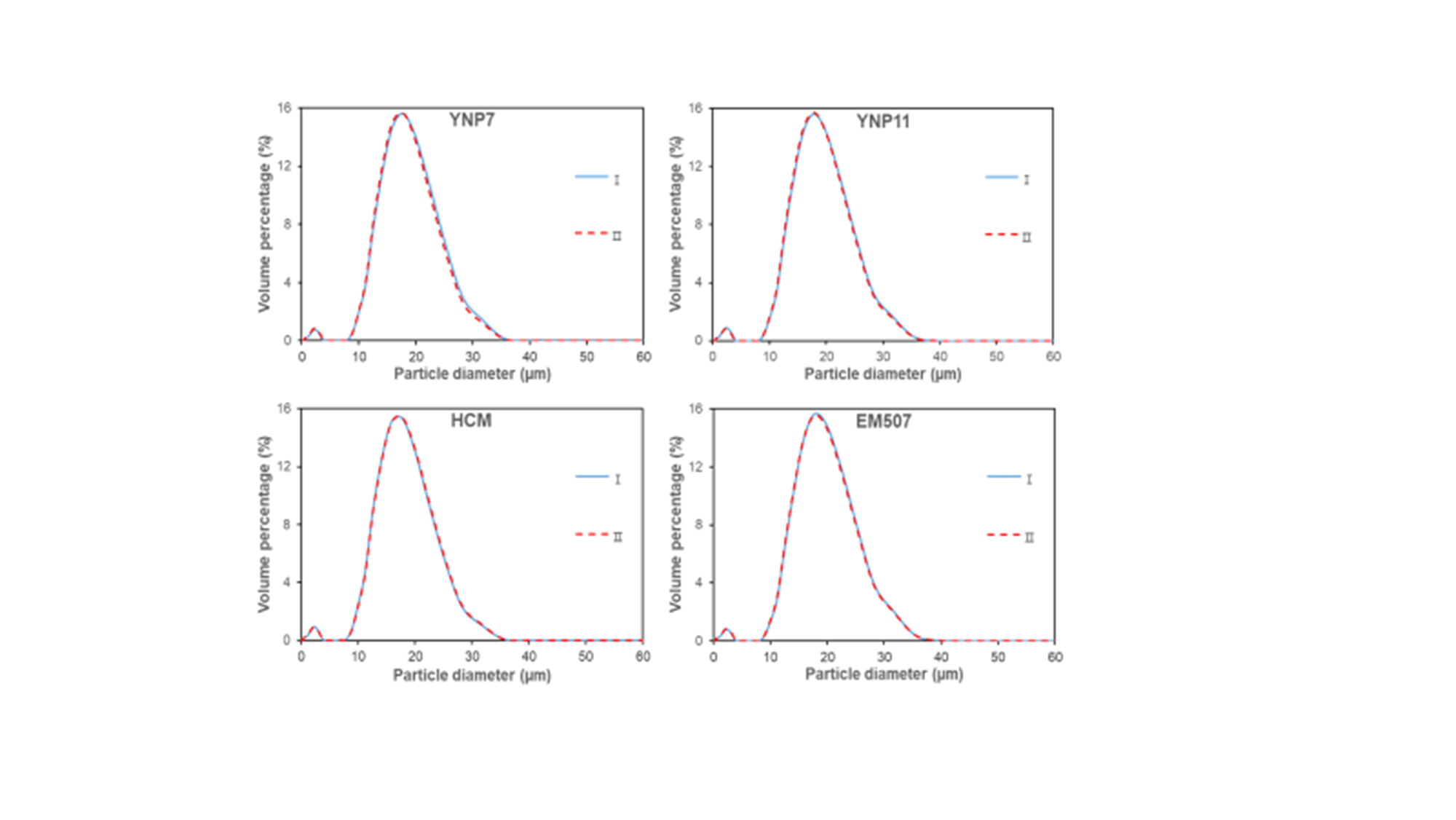

Supplement: S1 Fig — I, the sample of starch isolated from method 1; II, the sample of starch isolated from method 2. (TIF) [file pone.0216978.s001.tif]

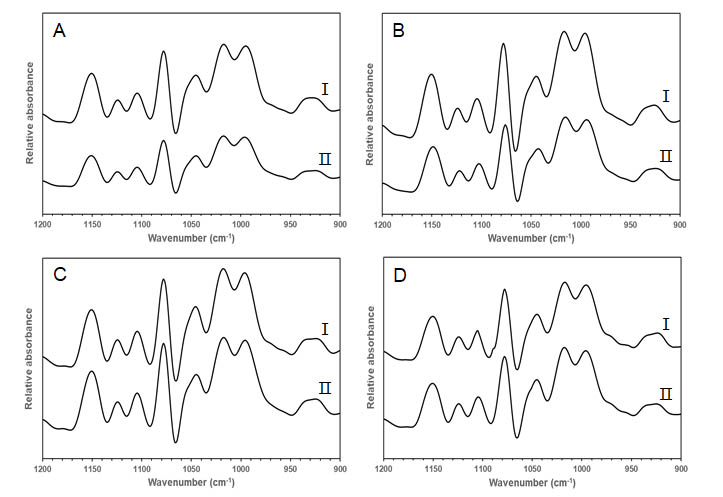

Supplement: S2 Fig — A: YNP7; B: YNP11; C: HCM; D: EM507. I, the sample of starch isolated from method 1; II, the sample of starch isolated from method 2. (JPG) [file pone.0216978.s002.jpg]

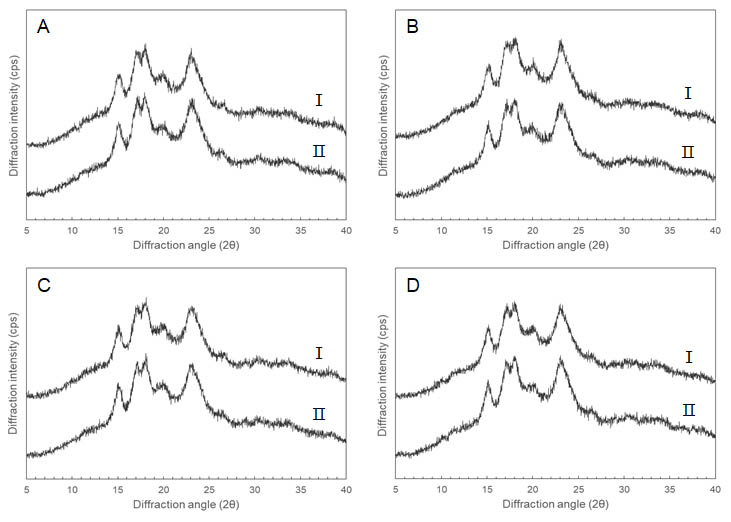

Supplement: S3 Fig — A: YNP7; B: YNP11; C: HCM; D: EM507. I, the sample of starch isolated from method 1; II, the sample of starch isolated from method 2. (JPG) [file pone.0216978.s003.jpg]
