# Supplementary material for: Comparison of pasting properties measured from the whole grain flour and extracted starch in barley (Hordeum vulgare L.)
Source: PLoS One. 2019 May 29;14(5):e0216978. doi: 10.1371/journal.pone.0216978 (PMC6541268; doi:10.1371/journal.pone.0216978)
Supplement: S1 Table — (DOCX) [file pone.0216978.s004.docx]

Table S1 RVA profile parameters measured of flour and starch from thirty barley varieties

| Varieties | PV (cP) | | TV (cP) | | BD (cP) | | FV (cP) | | SB (cP) | | PT (min) | | GT (℃) | |
| --- | --- | --- | --- | --- | --- | --- | --- | --- | --- | --- | --- | --- | --- | --- |
|  | Ⅰ | Ⅱ | Ⅰ | Ⅱ | Ⅰ | Ⅱ | Ⅰ | Ⅱ | Ⅰ | Ⅱ | Ⅰ | Ⅱ | Ⅰ | Ⅱ |
| Yangnongpi6 | 3862 | 4374 | 2684 | 2996 | 1178 | 1378 | 3907 | 4641 | 1223 | 1645 | 6.53 | 5.87 | 74.9 | 86.15 |
| Yangnongpi5 | 5337 | 4532 | 3120 | 2930 | 2217 | 1602 | 4752 | 4638 | 1632 | 1708 | 6.47 | 5.73 | 73.4 | 70 |
| Yangpi1 | 4372 | 4138 | 2552 | 2779 | 2020 | 1359 | 4040 | 4526 | 1488 | 1747 | 6.73 | 5.80 | 74.1 | 82.9 |
| Yangnongpi8 | 5142 | 4252 | 3294 | 2897 | 1848 | 1355 | 4947 | 4483 | 1653 | 1586 | 6.67 | 5.67 | 73.35 | 70.8 |
| Harrinonijo | 4744 | 4617 | 2882 | 3094 | 1862 | 1523 | 4656 | 5049 | 1774 | 1955 | 6.47 | 5.80 | 74.95 | 78.48 |
| Supi3 | 4620 | 3532 | 3056 | 2995 | 1364 | 537 | 4717 | 4289 | 1661 | 1294 | 6.80 | 6.40 | 81.25 | 87.75 |
| Yan99175 | 3883 | 3225 | 2425 | 2338 | 1458 | 887 | 3780 | 3863 | 1355 | 1525 | 6.60 | 6.20 | 81.2 | 87.7 |
| Hua11 | 4614 | 3940 | 2821 | 2756 | 1793 | 1184 | 4637 | 4564 | 1816 | 1808 | 6.60 | 6.07 | 77.35 | 80.25 |
| Hua22 | 4490 | 4787 | 2699 | 2863 | 1791 | 1924 | 4319 | 5365 | 1620 | 2502 | 6.67 | 5.87 | 75.7 | 84.5 |
| Haronanijo | 3565 | 3893 | 2419 | 2459 | 1146 | 1434 | 3299 | 4382 | 880 | 1923 | 6.53 | 5.73 | 74.15 | 85.35 |
| Rudong6109 | 3953 | 4126 | 2819 | 2964 | 1134 | 1162 | 3798 | 4538 | 979 | 1574 | 6.47 | 6.00 | 74.9 | 84.5 |
| Hong07-456 | 5145 | 3904 | 2833 | 2313 | 2312 | 1591 | 4932 | 3618 | 2099 | 1305 | 6.60 | 5.40 | 72.45 | 84.5 |
| Zhepi33 | 5208 | 4272 | 3131 | 2912 | 1877 | 1360 | 4784 | 4696 | 1653 | 1784 | 6.73 | 5.87 | 74.05 | 85.4 |
| Supi4 | 4906 | 4263 | 3219 | 3008 | 1687 | 1255 | 5305 | 4802 | 2086 | 1794 | 6.47 | 5.93 | 77.25 | 80.25 |
| Yunpi2 | 3906 | 4002 | 2553 | 2724 | 1353 | 1278 | 3571 | 4323 | 1018 | 1599 | 6.47 | 5.73 | 74.05 | 86.95 |
| Yunpi7 | 5568 | 4144 | 3356 | 2625 | 2212 | 1519 | 4922 | 4245 | 1566 | 1620 | 6.27 | 5.53 | 72.4 | 69.15 |
| Favorit | 5288 | 4557 | 3503 | 3194 | 1685 | 1363 | 5167 | 4825 | 1664 | 1631 | 6.40 | 5.80 | 74.05 | 70.85 |
| Zhumai5 | 5471 | 4179 | 3655 | 2537 | 1816 | 1642 | 5269 | 3626 | 1614 | 1089 | 6.27 | 5.27 | 71.65 | 82.9 |
| Huadamai7 | 3282 | 3624 | 2113 | 2747 | 1169 | 877 | 3324 | 4503 | 1211 | 1756 | 6.80 | 6.00 | 78.15 | 86.9 |
| Gairdner | 4426 | 4038 | 2877 | 2958 | 1549 | 1080 | 4092 | 4925 | 1215 | 1967 | 6.80 | 5.93 | 75 | 85.35 |
| Frankin | 5295 | 4213 | 3669 | 2956 | 1626 | 1257 | 5086 | 4652 | 1417 | 1696 | 6.40 | 6.00 | 74.85 | 80.25 |
| Yangnongpi9 | 4876 | 4520 | 2745 | 2509 | 2131 | 2011 | 4962 | 4253 | 2217 | 1744 | 6.60 | 5.40 | 73.2 | 81.3 |
| Yangnongpi10 | 4541 | 4473 | 2397 | 2370 | 2144 | 2103 | 4637 | 3813 | 2240 | 1443 | 6.27 | 5.20 | 71.65 | 82.9 |
| Yangsimai1 | 4414 | 4536 | 2812 | 2974 | 1602 | 1562 | 4444 | 4872 | 1632 | 1898 | 6.53 | 5.93 | 74.95 | 80.25 |
| Yangsimai3 | 4156 | 3690 | 2748 | 2826 | 1408 | 864 | 4172 | 4410 | 1424 | 1584 | 6.60 | 5.87 | 75.7 | 80.25 |
| Yangnongpi12 | 3960 | 4270 | 2529 | 2762 | 1631 | 1508 | 4071 | 4909 | 1542 | 2147 | 6.23 | 5.87 | 75.75 | 74.95 |
| Hu1154 | 3599 | 3434 | 2337 | 2343 | 1262 | 1091 | 3946 | 4350 | 1609 | 2007 | 6.53 | 6.20 | 81.2 | 87.8 |
| Yang0187 | 3797 | 3775 | 2156 | 2613 | 1641 | 1162 | 3760 | 4680 | 1604 | 2067 | 6.63 | 6.13 | 77.3 | 86.9 |
| Taixin9425 | 4871 | 5048 | 2957 | 3276 | 1914 | 1772 | 4682 | 5617 | 1725 | 2341 | 6.13 | 6.00 | 74.8 | 86.05 |
| Yangnongpi2 | 5255 | 4161 | 3357 | 2869 | 1898 | 1292 | 4959 | 4575 | 1602 | 1706 | 6.13 | 6.07 | 75 | 71.6 |

Ⅰ, the sample of grain flour; Ⅱ, the sample of starch isolated from method 2.
